# THE ROLE OF GERONTOLOGISTS IN ACHIEVING THE UN’S SUSTAINABLE DEVELOPMENT GOALS

**DOI:** 10.1093/geroni/igz038.298

**Published:** 2019-11-08

**Authors:** Carole Cox, Carole B Cox

**Affiliations:** Fordham University, New York, New York, United States

## Abstract

The Sustainable Development Goals (SDG’s) developed by the United Nations in 2015 are global benchmarks for all countries to meet by 2030 to ensure well-being and prosperity while protecting and promoting human rights and freedoms. The underlying pledge is that no one will be left behind Globally, older adults are one of the most vulnerable populations, suffering from poverty and poor health and little social protection. Social workers can play key roles in assuring that the concerns and interests and rights of older adults are recognized in the SDGs and in the policies developed to meet them. This paper focuses on 6 of the SDG’s that are most pertinent to the status and inclusion of older people and the implications they have for specific social work involvement.

